# Prospective Teachers’ Knowledge of Physical Activity in Children and Adolescents

**DOI:** 10.3390/healthcare12020236

**Published:** 2024-01-18

**Authors:** Sandra Milena Moreno-Lavaho, Jorge Pérez-Gómez, Irene Polo-Campos, Santiago Gómez-Paniagua, Jorge Rojo-Ramos

**Affiliations:** 1 Research Group Training in Movement, Universidad del Tolima, Tolima 730006299, Colombia; smmorenolv@ut.edu.co; 2 Health, Economy, Motricity, and Education (HEME) Research Group, Faculty of Sport Sciences, The University of Extremadura, 10003 Cáceres, Spain; 3 BioẼrgon Research Group, University of Extremadura, 10003 Cáceres, Spain; ipolocampos@gmail.com (I.P.-C.); jorgerr@unex.es (J.R.-R.)

**Keywords:** knowledge, physical activity, health, future teachers, CUAFYS-A, gender, age, physical activity level

## Abstract

Over the past few years, organizations around the world have tried to reach different populations with recommendations about physical activity (PA), due to this subject playing an important role in the phases of intention development and in preparation for it. Thus, the knowledge of future educators in the fields of health and PA is of vital importance when improving the levels of the latter in students. The objective of this research is to determine what knowledge future teachers have about health and PA, examining possible disparities according to the gender, age, and level of physical exercise of the participants. In addition, the psychometric properties of the instrument used were explored. A total of 321 Colombian university students from the Faculty of Education between the ages of sixteen and thirty-five participated in this study, providing sociodemographic information by filling out the CUAFYS-A questionnaire. Significant differences were found in the scale items according to sex and self-perception of being physically active. Also, the findings revealed a single-factor structure with nine items that had satisfactory reliability (α = 0.71; CR = 0.72) and excellent goodness-of-fit indices (RMSEA = 0.055 (90% CI (0.3, 0.8), RMSR = 0.02, CFI = 0.935, NNFI = 0.912, CMIN/DF = 1.97). Therefore, strategies and campaigns to promote PA knowledge in prospective teachers should be tailored according to gender and PA levels. Similarly, the CUAFYS-A questionnaire can be considered a valid and reliable instrument to identify the PA knowledge of future educators.

## 1. Introduction

Physical activity (PA) is defined as “any bodily movement produced by skeletal muscles that requires energy expenditure (...)” according to the World Health Organization (WHO) [1]. As a result, it is possible to differentiate between activities that are necessary for maintaining life and those whose main goal is to have fun, socialize, develop physical fitness, or even compete [2]. According to Devís [3], any intentional movement of the skeletal muscles that necessitates the use of energy and permits interaction with others and the environment is referred to as PA. Likewise, depending on the WHO [4], “a condition of complete physical, mental, and social well-being and not only the absence of disease or infirmity” is what is meant by the idea of health. The WHO emphasizes the crucial part that exercise plays in promoting healthy aging and a high quality of life [5], recognizing exercise as a planned, scheduled, repetitive subset of PA with the ultimate or intermediate goal of enhancing or maintaining physical fitness [6]. In this way, physical inactivity (PI) has developed into a public health issue, leading to a variety of diseases such as degenerative, cardiovascular, metabolic, and various forms of cancer [7]. However, a sizable part of adults (31%) and teenagers (80%) are currently categorized as being insufficiently active [8]. Thus, the WHO have made a tremendous effort to make the population aware of the PA recommendations that should be developed for good health [9].

In this context, for the population affected by sedentary habits, the college years are a crucial time for the formation of lifestyle habits, which can have a long-term effect on the development of chronic diseases [10]. When it comes to physical activity, college students are likewise less active than the average adult population [11]. For instance, research shows that over 70% of college students do not walk the suggested 10,000 steps a day [12]. Various reports and publications have identified universities and schools as places to raise awareness and educate students about healthy behavioral choices, including healthy dietary practices, regular PA, and weight management [13,14]. Also, the COVID-19 pandemic-related measures resulted in the complete shutdown of educational facilities, including higher education institutions [15], requiring the university population to adjust to a primarily virtual learning environment [16]. The pandemic’s impacts are especially alarming because, even before the epidemic, it has been observed that a significant portion of university students worldwide are physically inactive [17], even greater than the global age-standardized prevalence [11]. In addition, a recently published investigation that included a systematic review and meta-analysis found that this cohort spent an average of nine hours per day inactive, on average, during the previous ten years [18].

In this sense, the relationship between knowledge and actual PA has been the subject of studies that have yielded contradictory results [19], despite the fact that the knowledge and understanding of the guidelines are essential prerequisites for maintaining and/or complying with PA recommendations [20]. There are many factors that influence participation in PA, for example, men have higher levels of PA, and their greater participation in heavy work may be the fundamental cause [21]. Similarly, individuals who had completed college or higher had a greater probability of engaging in physical exercise compared to those who could not read and write, possibly because of their previous contact with physical education programs [22]. Also, according to various studies [23,24], the knowledge of PA recommendations was a predictor of the level of PA, and those who were aware of the recommendations performed much more PA than those who were not. Loughlan and Mutrie observed a significant increase in PA levels after providing research participants with general exercise and PA recommendations [25]. In view of the above, school educators have a crucial responsibility and influence on the PA levels of students. Teacher support has been identified as a positive predictor of student participation in PA [26], with low levels of teacher support being one of the main barriers to PA practice [27]. It has also been shown that teachers with PA knowledge are able to improve PA levels and fundamental movement skills better than other teachers [28]. To this end, research points to effective motivation as essential to achieve higher levels of PA [29], with teacher training programs being of great use in order to enhance the motivation of their students, increasing their intentions to be physically active [30].

As can be seen in numerous previous investigations, there are several questionnaires related to PA [22,31,32,33,34], but all of them focus on collecting information about PA levels or about factors affecting PA practice. However, few attempts have been made to understand the relationship between the knowledge about PA and PA practice [35], finding the imperative need to adapt physical exercise programs to generate this knowledge in the population and not only focus on physical and psychological improvements, so that there is greater adherence to PA. Given the low levels of PA in adolescents reported by the WHO, it is critical and important to have methods to evaluate and monitor the amount of knowledge that students and educators have about the guidelines on PA and health. With this knowledge, decision makers can put programs and policies into place that affect raising PA rates and enhancing efficacy. Similarly, the use of reliable and validated measurement methods to assess the understanding of PA recommendations seems to have a positive impact in certain populations [36], especially those with higher levels of PI. Nevertheless, Spanish-speaking communities around the world lack reliable instruments to collect information about the knowledge on PA recommendations, so that the measures and strategies implemented to improve them do not have prior information to be adapted to the target population. Along these lines, the Colombian university population lacks studies that analyze their current state of knowledge about the WHO recommendations. Due to all of the above, this research aims to evaluate the level of knowledge that students of the Faculty of Education Sciences of the University of Tolima have about PA recommendations for children and adolescents, analyzing the possible differences that exist in the student body according to gender, age, and their levels of PA. Likewise, the aim is to assess the construct validity, internal consistency, and reliability of the CUAFYS-A questionnaire [37] to analyze university students’ knowledge of international PA recommendations, making it one of the few scientific evaluation tools available.

## 2. Materials and Methods

### 2.1. Participants

The sample included 321 university students from the Faculty of Education Sciences of the University of Tolima (Colombia), with a population of 1934 enrolled students, using simple random sampling [38], with a margin of error of 0.05 and a confidence level of 95%. However, participants had to meet the following inclusion criteria: (a) be an active student of the Faculty of Educational Sciences of the University of Tolima, (b) accept participation through informed consent, and (c) complete the CUAFYS-A questionnaire [37]. Different age ranges were defined for the study of the participants, including the following: (1) from 16 to 19 years of age, representing the end of adolescence; (2) from 20 to 29 years of age, identified as the beginning of adulthood; and (3) from 30 years of age, as a turning point in which certain socioeconomic changes occur that influence PA levels and knowledge [39]. Table 1 defines the sociodemographic characteristics of the sample.

### 2.2. Instruments

For the sociodemographic characterization, the questionnaire included three questions on gender, age, and a perception question asking whether they considered themselves physically active. In addition, the CUAFYS-A questionnaire [37] was used. This instrument consists of nine questions (Appendix A, Table A1) and aims to assess the health literacy and PA knowledge of adults according to the 2010 WHO recommendations. The questionnaire consists of multi-response questions with a single answer, a Cronbach’s alpha reliability of 0.74, and direct scores between 0 and 9 as the maximum and minimum values, depending on whether the answer is correct (1 point) or incorrect (0 points). Lastly, three classifications are devised to exhibit the threshold values of the scale. Participants with less than 50% correct answers on the questionnaire fall into the first threshold, which represents poor knowledge; those with between 50% and 75% correct answers fall into the second threshold, which indicates sufficient knowledge; and those with more than 75% correct answers fall into the third threshold, which reflects good knowledge.

### 2.3. Procedure

This is a quantitative, non-experimental, descriptive, and cross-sectional study [40]. The use of an electronic questionnaire (Google Forms) was selected, because it allowed for the cost-saving storing of all responses in one database, a higher response rate, and the prevention of data loss. Before completing the CUAFYS-A questionnaire, respondents read and signed the informed consent form and received information on the objectives of this study, the rights of the participants, and the researcher’s contact information for any questions regarding this study. Subsequently, they proceeded to answer the questionnaire with an approximate duration of 10 min. All data were collected and used anonymously following the ethical standards of the 2008 Declaration of Helsinki of the World Medical Association [41], which promotes the dignity of persons engaged in health research and the protection of their welfare. In addition, this study was approved by the Bioethics Committee of the University of Extremadura (66/2020) on 13 July 2020. Data collection took place between September and October 2021.

### 2.4. Statistical Analysis

Initially, the program FACTOR v.10.10.02 (Rovira I Virgili University: Tarragona, Spain) was used to conduct the exploratory analysis (EFA) [42]. The components were extracted utilizing Promin rotation together with the robust unweighted least squares (RULS) method [43], presuming that there is an association between them. Owing to the characteristics of the data, a polychoric correlation matrix [44] was employed, and the optimal number of dimensions was determined using parallel analysis [45]. Also, as a means of determining sample adequacy, the Bartlett and Kaiser–Meyer–Olkin (KMO) tests of sphericity were employed [46]. Finally, the resulting structure was stripped of cross loads larger than 0.40, communalities less than 0.30, and loads less than 0.40 [47,48].

Later, the confirmatory factor analysis was then conducted using the AMOS v.26.0.0 program (IBM Corporation, Wexford, PA, USA) to assess construct validity. The model’s goodness of fit was evaluated using the following metrics: the root mean square error of approximation (RMSEA) [49], the root mean square of residuals (RMSRs) [50], the comparative fit index (CFI) [51], the non-normed fit index (NNFI) [52], and the chi-square per degree of freedom ratio (CMIN/DF) [53], taking into account that the RMSEA should be between 0.01 and 0.05, the RMSR below 0.08, CFI and NNFI above 0.9, and the CMIN/DF below 2 [54]. Furthermore, Cronbach’s alpha was used as a measure of internal consistency, considering values above 0.7 as satisfactory [55], as well as the inter-item correlations, which should show values between 0.15 and 0.50 [56]. Finally, composite reliability (CR) was selected as a measure of the reliability of the instrument whose cutoff value is 0.7 [57].

For the purpose of analyzing the responses of the participants, the data were processed using the IBM SPSS statistical program for MAC (Chicago, IL, USA), version 23. First, the assumption of normality in the data distribution of the continuous variables was investigated using the Kolmogorov–Smirnov test. The adoption of nonparametric statistical tests followed the confirmation that this presumption was incorrect. The chi-square test was utilized to assess the item responses by differentiating them by gender, age, and PA level after the frequencies of the questionnaire responses were made.

## 3. Results

### 3.1. Exploratory Factor Analysis

Initially, the RULS approach was able to determine a monofactorial structure for the questionnaire through the explained variance based on eigenvalues [58] and the reliability of expected a posteriori scores (EAPs) [59], finding a score of 3.29 for the eigenvalue of this single factor as well as a variance proportion of 0.36. Additionally, positive results were obtained from the sampling adequacy indicators (KMO test = 0.79855 and Bartlett’s test = 814.9; df = 36; *p* = 0.000), confirming the feasibility of the EFA (refer to Appendix A, Table A2 for the polychoric correlation matrix). Subsequently, and due to the unidimensional nature of the scale, no rotation mechanism was chosen to run the EFA, resulting in a load matrix of nine items (Table 2).

### 3.2. Confirmatory Factor Analysis

**Figure 1:**
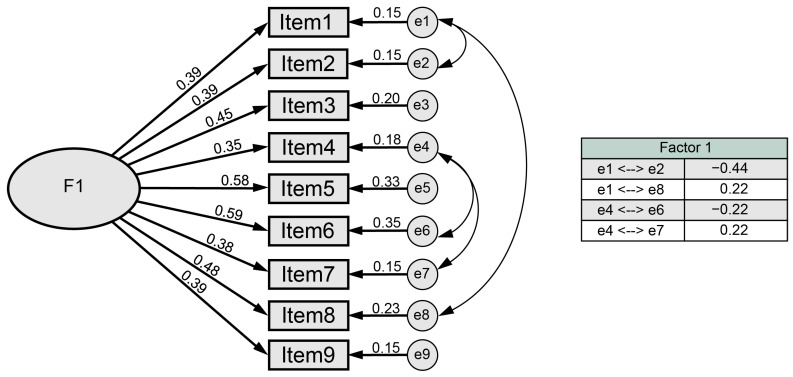
Factorial model of the CUAFYS-A questionnaire.

The initial configuration obtained in the CFA resulted in unacceptable goodness-of-fit indices for the established cut-off values (RMSEA = 0.088 (90% CI (0.069–0.107), RMSR = 0.027, CFI = 0.806, NNFI = 0.753, CMIN/DF = 2.845). To solve this problem, and after observing that all the items have adequate factorial characteristics, several error terms were correlated (see Figure 1). Once the errors were correlated, goodness-of-fit indices could be defined as optimal (RMSEA = 0.055 (90% CI (0.3, 0.8), RMSR = 0.02, CFI = 0.935, NNFI = 0.912, CMIN/DF = 1.97), demonstrating excellent construct validity.

As for the internal consistency of the instrument, satisfactory values (>0.70) were obtained in reference to Cronbach’s alpha (α = 0.71), being also unable to improve despite the elimination of some items from the questionnaire. Additionally, inter-item correlations demonstrated appropriate scores, since all of them were among the values described by the scientific literature (Table 3), except for item 2, where some contradictory scores were found. Finally, CR also showed satisfactory values in the proposed model (CR = 0.72) in terms of reliability.

### 3.3. Descriptive Results

In general terms (Table 4), the sample has a very low level of knowledge of PA recommendations (Me = 2), with only 4 participants getting at least 50% of the questions correct. In terms of gender, no statistically significant differences (*p* = 0.16) were observed between the two groups, even though women had higher scores. In the same way, these results are extrapolated to age, where the group between 30 and 35 years of age is the most accurate; however, no significant differences were obtained when compared to the other groups (*p* = 0.15). On the other hand, those participants who did not consider themselves physically active obtained better results than their peers who were physically active, with the statistic showing significant differences (*p* < 0.01).

Table 5 shows the number of times that each answer of the CUAFYS-A questionnaire was selected by the participants. It is worth noting that the only items with more than 50% correct answers are 1 and 8, while, in all the others, there are not even 25% right answers. Similarly, most of the participants’ responses were found in the third option of the items: “Don’t know”.

Table 6 presents descriptive information for each question of the CUAFYS-A questionnaire based on the numerical value (N) presented by each response option according to the gender of the students. In this sense, statistically significant differences were observed in item 4, because only three female participants answered “Disagree”, with the minimum expected number of responses being eight.

Table 7 shows the results of each question of the CUAFYS-A questionnaire according to the frequencies found in each response option when observing the different age groups. It is clear that no major discrepancies were discovered, since none of the questions yielded statistical significance.

Table 8 provides descriptive information on the frequencies obtained in each option of each of the CUAFYS-A items according to university students’ self-perception of whether they are physically active or not. Significant differences were observed for items 6 and 9, with a significance of *p* < 0.01, and items 1, 3, 7, and 8, with a significance of *p* < 0.05.

## 4. Discussion

This study was born out of two needs expressed by the literature: (1) to discover the level of knowledge that university students have about health and PA, and (2) to define a valid and reliable instrument to gather information on the knowledge of PA recommendations in the university population. For this purpose, the questions of the CUAFYS-A instrument were analyzed, taking into account that gender, age, and level of PA could influence knowledge. In addition, the construct validity, internal consistency, and reliability of the instrument were analyzed, resulting in a nine-item single-factor structure with excellent goodness-of-fit indices and satisfactory reliability indicators. Also, in the present investigation, it was observed that, in general, future male teachers have a higher average knowledge of the WHO recommendations on PA and health than female future teachers. Also, people between 20 and 29 years of age are the most aware of these recommendations, and those university students who have higher levels of PA report higher scores.

In terms of gender, it is generally women who perform less PA [60,61,62], with a frequency between 10 min and 1 h, while men usually perform at least 1 h of sports practice [63]. This trend has already been noted by Han and colleagues [64], who identified a reduction in PA levels during the transition from high school to university, which was more pronounced in female students. Likewise, not only the lack of time seems to be a relevant factor, in the case of women, as other aspects such as physical social anxiety associated with body image, exhaustion, or laziness or the environment and a lack of facilities are found to be the primary causes of physical inactivity [65]. However, studies focusing primarily on PA knowledge show mixed results. According to the study by Jáuregui-Lobera and Oliveras López [66], there were no statistically significant gender differences in any of the questions about the knowledge of PA. Likewise, Keating et al. [67] explored the knowledge levels of U.S. university students without finding significant differences in terms of gender. This fact has also been exposed by more recent research, which has pointed out that there are no gender differences in terms of the knowledge of PA recommendations [68]. In contrast, in a more recent research by Gómez-Mazorra et al. [60], significant differences were obtained, with a higher mean for the male gender. Similarly, Plotnikoff et al. [69] noted gender differences in the knowledge of the guidelines in a large Canadian population.

On the other hand, it has been shown that there are no significant differences in terms of student age in relation to PA and health knowledge. No significant variations in age were found by Práxedes et al. [70], which supports this as well. However, a number of studies suggest that PA levels fall as children enter adolescence [71], with this decline being more pronounced in females than in boys [72]. Adolescents are one of the population groups who do not prioritize their health needs; also, their unhealthy behaviors, developed at a young age, lead to major health issues as adults, putting them at a higher risk in terms of their physical, social, and psychological aspects [73]. In maturity, this lower trend persists, demonstrating the gradual renunciation of this lifestyle practice over time [74,75]. Similar to this, a cross-sectional research of students from high schools and universities revealed that university students had lower PA levels [76,77]. However, it has also been discovered that there are higher levels of PA practice in those who have completed their university education [78] and in those who are over the age of 21 [79]. It follows that they are more informed about the advantages of exercise and how it improves their health, as the results of this study seem to indicate.

Considering the self-perception of future teachers about being physically active, those with a positive self-perception showed better results. This issue was also observed by Knox and coworkers [80], who found that a failure to comply with physical activity recommendations was a strong predictor of a lack of knowledge of them. Similarly, another study in Africa [81] showed a positive association between the participation in PA and a good knowledge of PA recommendations. Another study conducted among Chinese university students found almost identical results to the present investigation, reporting that only 4% of the university students were aware of the recommendations and that they showed significantly higher PA levels and emphasizing the low prevalence of this knowledge in future physical education teachers (around 14%) [68]. This trend has been confirmed by an international study that included university students from 23 countries, which found a clear relationship between PI and a low knowledge of PA recommendations, although this was mostly found in men [17]. The explanation for these results is multifactorial, as other studies have already pointed out [82], since interventions that provide information on PA recommendations do not directly improve PA levels but do improve the intention to practice PA [83]. In addition, the positive association between knowledge and practice may be mainly due to the fact that they share common mediators such as ethnicity, level of education, economic status, or their health functionality [84].

### Limitations and Future Lines of Research

As in other studies, this research also has a series of limitations. First, given that only university students were included in the sample, there are factors that could have influenced the results obtained, such as age and its grouping by tens, sociodemographic characteristics, and the students’ school grade. Also, the division into age ranges, although it obeyed maturity stages throughout the life cycle, only collected 11 responses in the group over 30 years of age, so that comparisons between groups may not yield conclusive results. Similarly, sociodemographic information that could be interesting for this study, such as academic year, field of study, or nutritional habits, was not widely collected from the participants. Finally, it should be noted that there are very few previous studies that analyze the knowledge that university students have about health and PA. Rather, whether they are physically active or inactive is analyzed. On the other hand, possible future lines of research would be to extend the sample to a national level, instead of carrying out the experiment in only one faculty, therefore omitting the criterion that it has to be exclusively students from Tolima, to choose a more appropriate grouping method for age in case this could have contributed to the results, analyzing whether, in this case, age has an influence and whether women continue to be less active than men. Likewise, it would be interesting to include questions in the scales whose focus of analysis is lifestyles and various nutritional issues. In this case, knowing the reasons would be helpful, as they may be not only due to work, academic, and/or family tasks but also due to a lack of motivation. Consequently, it is essential to reach an agreement with other researchers in different communities to collect all the necessary data. In the same way, it could be of interest to promote longitudinal research after providing different types of education on PA recommendations to university students, exploring the sociodemographic factors that mediate them and which interventions have a greater impact in both the short and long term. Furthermore, it should not be forgotten that it is essential to train teachers using innovative and motivating methodologies, so that university students have carried them out before reaching the university stage, so that they can perform PA correctly and obtain all the psychological, physical, and social benefits that it brings to the health and well-being of individuals.

## 5. Conclusions

First, the CUAFYS-A questionnaire can be considered a valid and reliable instrument to analyze the knowledge about PA recommendations in future teachers. Also, this study has shown that men and those people that are physically more active showed a higher knowledge of health and PA recommendations.

## Figures and Tables

**Table 1:** The sample’s sociodemographic composition (N = 321).

Variables	Categories	N	%
Gender	Male	166	51.7
	Female	155	48.3
Age	16–19 years	108	33.6
	20–29 years	202	63.0
	30–35 years	11	3.4
Do you consider yourself physically active?	Yes	199	62.0
	No	122	38.0

N: number, %: percentage.

**Table 2:** Scale’s loading matrix.

Items	Load	Communality
1	0.469	0.32
2	0.417	0.374
3	0.539	0.39
4	0.446	0.399
5	0.76	0.577
6	0.62	0.385
7	0.481	0.421
8	0.591	0.449
9	0.422	0.387

**Table 3:** Inter-item correlations and reliability if item is deleted.

Items	1	2	3	4	5	6	7	8	9	Cronbach’s Alpha If Deleted
1	0.33									0.68
2	0.05	0.36								0.69
3	0.22	0.08	0.45							0.68
4	0.16	0.25	0.17	0.34						0.69
5	0.18	0.25	0.28	0.18	0.29					0.67
6	0.19	0.22	0.27	0.16	0.37	0.44				0.67
7	0.30	0.09	0.21	0.33	0.16	0.21	0.57			0.68
8	0.37	0.13	0.29	0.15	0.29	0.29	0.23	0.39		0.67
9	0.17	0.26	0.16	0.16	0.22	0.23	0.15	0.16	0.59	0.69

This table describes the variances of the items (diagonal) and the correlations between them (below the diagonal).

**Table 4:** Descriptive analysis of the total score of the items according to gender, age, and self-perceived PA level.

	Variables									
Total Score Me (IQR)	Gender Me (IQR)			Age Me (IQR)				Physically Active Me (IQR)		
	Male	Female	*p*	16–19	20–29	30–35	*p*	Yes	No	*p*
2 (2)	2 (2)	2 (2)	0.16	2 (2)	2 (2)	3 (2)	0.15	2 (2)	2 (1)	<0.01 **

Note: Me = median value, IQR = interquartile range. Differences are significant at ** *p* < 0.01.

**Table 5:** Frequencies of responses to the questionnaire.

Items	Answers		
	Strongly Agree	Disagree	Don’t Know
	N (%)	N (%)	N (%)
1	34 (10.6)	**208 (64.8)**	79 (24.6)
2	**24 (7.5)**	49 (15.3)	248 (77.3)
3	33 (10.3)	**75 (23.4)**	213 (66.4)
4	**27 (8.4)**	17 (5.3)	277 (86.3)
5	**21 (6.5)**	19 (5.9)	281 (87.5)
6	**33 (10.3)**	50 (15.6)	238 (74.1)
7	**56 (17.4)**	106 (32.9)	159 (49.4)
8	35 (10.9)	**180 (55.9)**	106 (32.9)
9	**54 (16.8)**	71 (22.0)	196 (60.9)

Note: N = number; % = percentage. Each score obtained in the dimensions is based on a Likert scale (1–3) with a single correct answer marked in bold.

**Table 6:** Descriptive analysis and gender-specific variations of the questionnaire items.

Items	Answers	Gender			
		Total	Male	Female	
		N	N	N	*p*
1	Strongly agree	34	23	11	0.11
	Disagree	**208**	**101**	**107**	
	Don’t know	79	42	37	
2	Strongly agree	**24**	**10**	**14**	0.24
	Disagree	49	30	19	
	Don’t know	248	126	122	
3	Strongly agree	33	17	16	0.84
	Disagree	**75**	**41**	**34**	
	Don’t know	213	108	105	
4	Strongly agree	**27**	**15**	**12**	0.03 *
	Disagree	17	14	3	
	Don’t know	277	137	140	
5	Strongly agree	**21**	**10**	**11**	0.37
	Disagree	19	7	12	
	Don’t know	281	149	132	
6	Strongly agree	**33**	**16**	**17**	0.59
	Disagree	50	23	27	
	Don’t know	238	127	111	
7	Strongly agree	**56**	**26**	**30**	0.68
	Disagree	106	56	50	
	Don’t know	159	84	75	
8	Strongly agree	35	19	16	0.51
	Disagree	**180**	**88**	**92**	
	Don’t know	106	59	47	
9	Strongly agree	**54**	**24**	**30**	0.42
	Disagree	71	40	31	
	Don’t know	196	102	94	

Note: N = number. Differences are significant at * *p* < 0.05. Each score obtained in the dimensions is based on a Likert scale (1–3) with a single correct answer marked in bold.

**Table 7:** Descriptive analysis and age-specific changes to the survey items.

Items	Answers	Age				
		Total	16–19	20–29	30–35	
		N	N	N	N	*p*
1	Strongly agree	34	11	21	2	0.83
	Disagree	**208**	**73**	**128**	**7**	
	Don’t know	79	24	53	2	
2	Strongly agree	**24**	**8**	**15**	**1**	0.71
	Disagree	49	18	31	0	
	Don’t know	248	82	156	10	
3	Strongly agree	33	10	22	1	0.74
	Disagree	**75**	**28**	**43**	**4**	
	Don’t know	213	70	137	6	
4	Strongly agree	**27**	**8**	**18**	**1**	0.13
	Disagree	17	2	13	2	
	Don’t know	277	98	171	8	
5	Strongly agree	**21**	**6**	**15**	**0**	0.60
	Disagree	19	5	14	0	
	Don’t know	281	97	173	11	
6	Strongly agree	**33**	**13**	**19**	**1**	0.78
	Disagree	50	14	35	1	
	Don’t know	238	81	148	9	
7	Strongly agree	**56**	**20**	**34**	**2**	0.98
	Disagree	106	37	65	**4**	
	Don’t know	159	51	103	5	
8	Strongly agree	35	11	23	1	0.45
	Disagree	**180**	**62**	**109**	**9**	
	Don’t know	106	35	70	1	
9	Strongly agree	**54**	**18**	**32**	**4**	0.43
	Disagree	71	26	44	1	
	Don’t know	196	64	126	6	

Note: N = number. Each score obtained in the dimensions is based on a Likert scale (1–3) with a single correct answer marked in bold.

**Table 8:** Descriptive analysis and modifications to the survey items based on PA levels.

Items	Answers	Do You Consider Yourself Physically Active?			
		Total	Yes	No	
		N	N	N	*p*
1	Strongly agree	34	14	20	0.01 *
	Disagree	**208**	**129**	**79**	
	Don’t know	79	56	23	
2	Strongly agree	**24**	**12**	**12**	0.12
	Disagree	49	36	13	
	Don’t know	248	151	97	
3	Strongly agree	33	14	19	0.03 *
	Disagree	**75**	**45**	**30**	
	Don’t know	213	140	73	
4	Strongly agree	**27**	**15**	**12**	0.06
	Disagree	17	15	2	
	Don’t know	277	169	108	
5	Strongly agree	**21**	**12**	**9**	0.15
	Disagree	19	8	11	
	Don’t know	281	179	102	
6	Strongly agree	**33**	**12**	**21**	<0.01 **
	Disagree	50	28	22	
	Don’t know	238	159	79	
7	Strongly agree	**56**	**30**	**26**	0.03 *
	Disagree	106	59	47	
	Don’t know	159	110	49	
8	Strongly agree	35	17	18	0.02 *
	Disagree	**180**	**106**	**74**	
	Don’t know	106	76	30	
9	Strongly agree	**54**	**24**	**30**	<0.01 **
	Disagree	71	51	20	
	Don’t know	196	124	72	

Note: N = number. Differences are significant at ** *p* < 0.01; * *p* < 0.05. Each score obtained in the dimensions is based on a Likert scale (1–3) with a single correct answer marked in bold.

## Data Availability

The datasets used during the current study are available from the corresponding author on reasonable request.

## Appendix A

**Table A1:** CUAFYS-A questionnaire items.

	Items	Strongly Agree	Disagree	Don’t Know
1	“Children from 5 to 17 years old should do vigorous activities (those that involve effort) at most 3 times a week (playing soccer, basketball, swimming...)”			
2	“Children from 5 to 17 years of age should do activities that involve bone strain (PA being understood as that which aims to increase strength at certain points of the bones of the locomotor apparatus), for example, running, jumping, swimming or lifting weights.”			
3	“In children aged 5 to 17 years, doing more than 1 h of PA every day (such as brisk walking, cycling, swimming...) can be detrimental to their health.”			
4	“According to WHO in children and young people, PA is considered to be: playing, sports, travel, recreational activities, PA or scheduled exercises (with family, at school or in their daily life).”			
5	“In children aged 5 to 17 years, doing more than one hour a day of PA (such as brisk walking, cycling, swimming) can be beneficial to their health.”			
6	“Children aged 5 to 17 years should do at least 1 h a day of PA (such as brisk walking, cycling, swimming...) every day.”			
7	“According to WHO, PA in children and young people is considered to be only: sports, physical education (PE) or scheduled exercise (with family, at school or in their daily life).”			
8	“Children aged 5 to 17 years should do no more than 1 h a day of PA (brisk walking, cycling, swimming...).”			
9	“Children aged 5 to 17 years should do vigorous activities (those involving an effort of 7–8 out of 10 on a scale of 0 to 10) at least 3 times a week (playing soccer, basketball, swimming).”			

**Table A2:** Polychoric correlation matrix extracted from the EFA.

Items	1	2	3	4	5	6	7	8	9
1	1.00								
2	−0.04	1.00							
3	0.28	0.05	1.00						
4	0.22	0.41	0.26	1.00					
5	0.25	0.43	0.44	0.29	1.00				
6	0.24	0.33	0.36	0.06	0.59	1.00			
7	0.40	0.10	0.28	0.47	0.22	0.26	1.00		
8	0.48	0.14	0.39	0.14	0.43	0.35	0.30	1.00	
9	0.17	0.38	0.13	0.21	0.36	0.30	0.10	0.19	1.00
